# Stem cells and repair of lung injuries

**DOI:** 10.1186/1465-9921-5-6

**Published:** 2004-07-20

**Authors:** Isabel P Neuringer, Scott H Randell

**Affiliations:** 1 Assistant Professor, Division of Pulmonary and Critical Care Medicine and Cystic Fibrosis/Pulmonary Research and Treatment Center, The University of North Carolina School of Medicine, Chapel Hill, North Carolina, USA; 2 Assistant Professor, Division of Pulmonary and Critical Care Medicine, Cystic Fibrosis/Pulmonary Research and Treatment Center and Department of Cellular and Molecular Physiology, The University of North Carolina School of Medicine, Chapel Hill, North Carolina, USA

**Keywords:** lung hypoplasia, respiratory distress syndrome, chronic lung disease of prematurity, pulmonary emphysema, pulmonary fibrosis, bronchiolitis obliterans, cystic fibrosis, asthma, lung cancer

## Abstract

Fueled by the promise of regenerative medicine, currently there is unprecedented interest in stem cells. Furthermore, there have been revolutionary, but somewhat controversial, advances in our understanding of stem cell biology. Stem cells likely play key roles in the repair of diverse lung injuries. However, due to very low rates of cellular proliferation *in vivo *in the normal steady state, cellular and architectural complexity of the respiratory tract, and the lack of an intensive research effort, lung stem cells remain poorly understood compared to those in other major organ systems. In the present review, we concisely explore the conceptual framework of stem cell biology and recent advances pertinent to the lungs. We illustrate lung diseases in which manipulation of stem cells may be physiologically significant and highlight the challenges facing stem cell-related therapy in the lung.

## Introduction

According to Greek mythology, the immortal Prometheus stole fire from the Gods as a gift for humankind. As punishment, he was shackled to a rock, whereupon each day for 30,000 years an eagle consumed as much of his liver as would regenerate. There is some debate whether the eagle ate his liver or heart, but what if the bird had a taste for lung? And what if Prometheus was a mere mortal?

Analogous to Prometheus and the eagle, the ambient air-exposed lung is subject to an array of potentially damaging agents, including chemical oxidants and proteolytic enzymes. Presumably, daily oxidant and protease wear and tear on structural components such as elastin and collagen contributes to inevitable age-related declines in pulmonary function in normal individuals [1,2]. Acute and chronic lung disease, or its treatment with oxygen and positive pressure ventilation, may further damage lung tissue in excess of the capacity for orderly repair, resulting in characteristic pathologic changes including tissue destruction or fibrotic scarring [3-5]. But what determines the lungs' capacity for repair? Certainly, one factor must be the ability of stem cells to proliferate and differentiate to replace damaged cells and tissues. As discussed later in this review, the traditional view is that, during development, self-renewing tissues are imbued with resident, tissue-specific stem cells, so-called adult somatic stem cells. However, recent but highly controversial evidence suggests that stem cells from one type of tissue may generate cells typical of other organs. In this fashion, circulating cells derived from bone marrow may augment resident stem cells, and we comprehensively review such data from lung. Finally, there is great hope that embryonic stem cells, embryonic germ cells, or even adult somatic stem cells can be engineered as an unlimited source of cells to enhance organ-specific repair or replace lost tissues. Below, we concisely review stem cell biology, focusing on recent findings relevant to the lungs. Diseases in which alterations in stem cells contribute to lung dysfunction are discussed, as are the challenges facing the nascent field of pulmonary regenerative medicine.

## Embryonic and adult (somatic) stem cells

**Figure 1:**
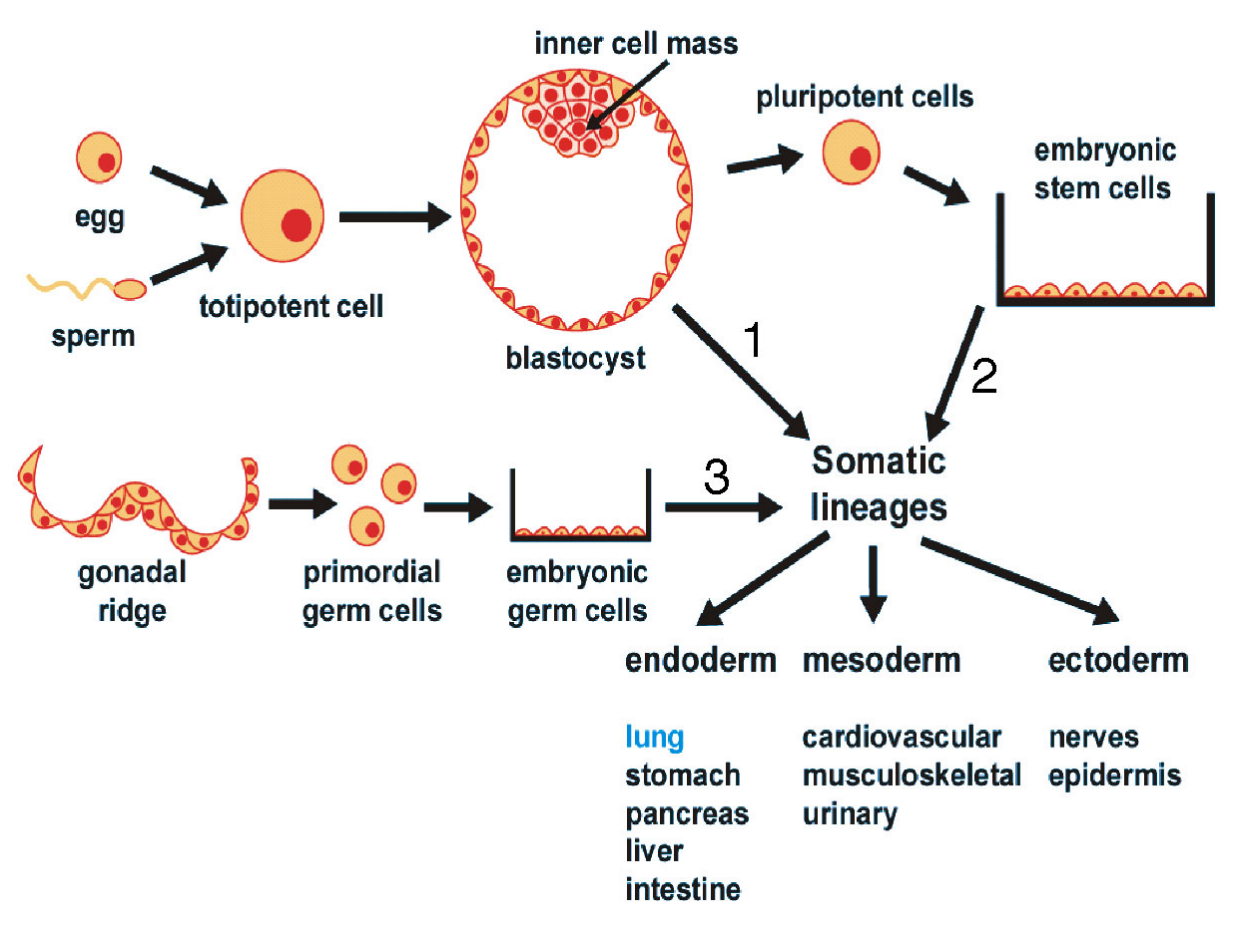
*Cell lineage determination during embryogenesis and generation of pluripotent embryonic cells*. The three primary germ layers form during normal development (path 1). Embryonic stem cells from the inner cell mass (path 2) or embryonic germ cells from the gonadal ridge (path 3) can be cultured and manipulated to generate cells of all three lineages.

For links to more in-depth information on general principles in stem cell biology, a comprehensive glossary, and the latest updates in this quick moving field, the reader is referred to the International Society for Stem Cell Biology . During embryonic development, the inner cell mass of the blastocyst forms three primary germ layers, which generate all fetal tissue lineages (reviewed in [6], illustrated in Figure 1, path 1). Embryonic stem cells (derived from the blastocyst inner cell mass), or embryonic germ cells (derived from the gonadal ridge), when cultured on embryonic mouse fibroblast feeder cell layers in the presence of a differentiation-suppressing cytokine (leukemia inhibitory factor), proliferate indefinitely and remain pluripotent. Manipulation of culture conditions can coax the cells to undergo differentiation characteristic of many tissue types (Figure 1, paths 2 and 3). Theoretically, pluripotent embryonic cells can serve as an unlimited resource for therapeutic applications [7,8].

**Figure 2:**
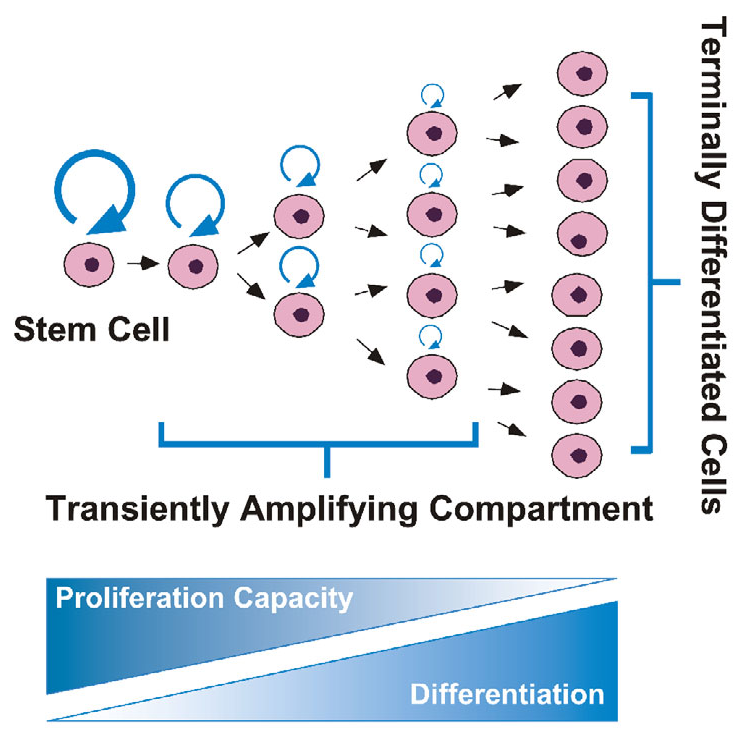
*Traditional view of cell lineage in adult renewing tissues*. Organ-specific (somatic) stem cells generate characteristic cell types through a linear set of commitment and differentiation steps. Arrow thickness represents self-renewal potential.

General principles of tissue renewal by adult stem cells have been reviewed recently [9] and can be summarized as follows. The traditional view of cell lineages is that adult somatic stem cells maintain cell populations in adult tissues. The adult lung falls into the category in which cell proliferation is very low in the normal steady-state but can be induced dramatically by injury (see [10,11] for recent reviews of lung stem cells). The conditional nature of lung cell proliferation complicates the search for lung stem cells. Cell lineages are much better understood in continuously proliferating tissues such as the gut, skin and hematopoietic system (reviewed in [12-14], respectively). The long-standing view, developed from these other organs, is that stem cells reside in well-protected, innervated, and vascularized niches that provide cues regulating cell fate decisions such as proliferation, migration, and differentiation [15]. Adult stem cells are capable of abundant self-renewal and can also generate the specific cell lineages within the tissue compartment (Figure 2). Proportional to tissue needs, stem cells may undergo asymmetric cell division, in which they generate one stem cell and a committed progenitor. The capacity for self-renewal decreases progressively as committed progenitors differentiate. The wisdom of the body is to conserve stem cells. They cycle infrequently and the majority of cell replacement is accomplished by committed progenitors within the so-called transiently amplifying compartment. Eventually, individual cells become incapable of further cell division. In tissues, there are specific temporal and spatial hierarchic relationships between stem cells in their niches and their differentiated progeny. Within this axis, cell proliferation, migration, differentiation, function, death, and removal are tightly regulated to maintain tissue homeostasis.

## Cell compartments in the lung and functional integration

**Figure 3:**
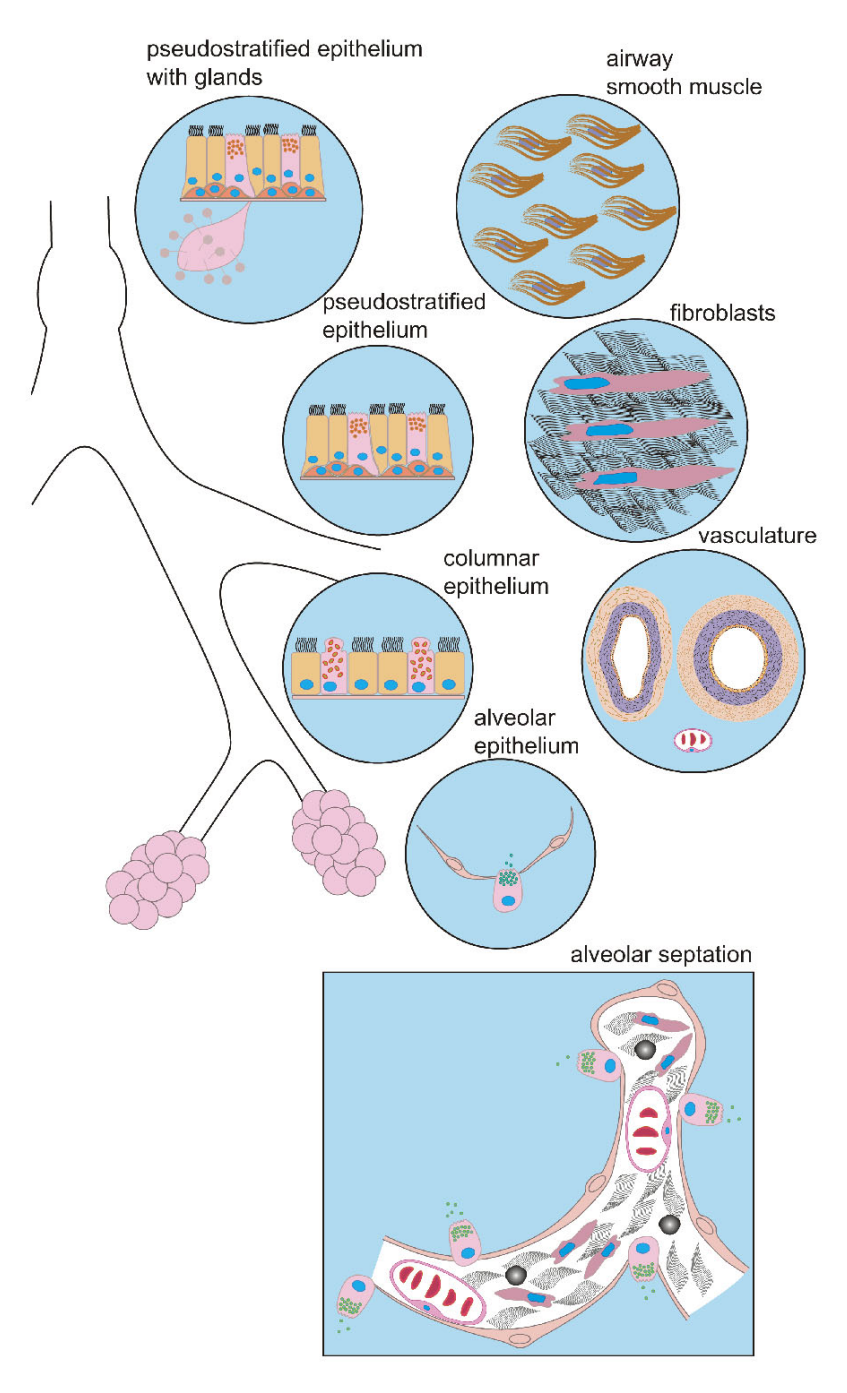
*Stem cell compartments in the lungs*. The endoderm-derived epithelium can be subdivided into at least 4 types whereas smooth muscle, fibroblasts, and vascular cells are derived from mesoderm. The coordinated interaction of multiple cell types, including alveolar epithelium, interstitial fibroblasts, myofibroblasts and pulmonary endothelium, is necessary to form alveolar septa.

In the architecturally complex lung, cells of multiple germinal lineages interact both during morphogenesis and to maintain adult lung structure. Even within derivatives of a single germ layer, cells become subdivided into separate cell lineage "zones". For example, the endoderm generates least four distinct epithelial regions, each with a different cellular composition (Figure 3). Additional cell types, including airway smooth muscle, fibroblasts, and the vasculature, are derived from mesoderm. Airway and alveolar architecture, and in turn, function, result from interaction among epithelium, smooth muscle, fibroblasts, and vascular cells, all within an elaborate structural matrix of connective tissue. The complexity of even this oversimplified view, which omits pulmonary neuroepithelial cells and bodies, innervation, and classical hematopoietically-derived cells such as dendritic cells, mast cells, and macrophages, has hindered identification of lung stem cells and patterns of cell migration during tissue renewal. Nevertheless, the prevailing view is that airway basal and Clara cells and alveolar type II cells serve as epithelial progenitors [11,16-19]. Cell lineages in the mesodermal compartments remain less well understood.

## Stem cell plasticity and the lung

Recent studies challenge the view that tissues are maintained solely by organ-specific stem cells. There is evidence that adult stem cells from a variety of sources can generate not only their own lineages, but those of other tissues, sometimes crossing barriers of embryonic derivation previously thought impenetrable [20,21,8]. There are a few controversial reports that adult stem cells from outside the bone marrow may reconstitute the hematopoietic system, but most of the evidence flows in the other direction- namely, that cells from the bone marrow can generate diverse non-hematopoietic cell types. Both experimental studies in animals and human clinical studies, summarized in Table 1, provide evidence for, and against, circulatory delivery of lung progenitor cells. While bone marrow-derived cells, such as alveolar macrophages, dendritic cells, mast cells, and lymphocytes, normally migrate to the lung, the surprise in the recent literature is that under certain circumstances circulating cells can apparently generate lung resident cells, including epithelial, endothelial, and myofibroblast cells. The technical approach towards identification of these cells is often technically challenging and involves co-localization of a donor cell marker, for example, the Y chromosome, in sex-mismatched transplantation, or a genetically engineered marker in mouse experiments, and proteins characteristic of the differentiated cell type in the lung, for example, keratin in epithelial cells or collagen in fibroblasts. As discussed below, the results are highly variable and often contradictory, depending on factors including the starting cell population, the methods for marker detection, and the amount of injury to the lung.

**Table 1:** Evidence for, and against, circulating progenitor cell generation of non-hematopoietic lung cell types.

**Study Type**	**Disease or Model**	**Tissue of Origin**	**Lung Cell Type Formed / Frequency**	**Method of Detection**	**Ref.**
Animal, *in-vivo*	BMT	MSC	Undefined mesenchymal cells / occasional	PCR for collagen gene marker	[30]
Animal, *in-vivo*	Bleomycin fibrosis	MSC	Type I pneumocytes / rare	β galactosidase protein	[23]
Animal, *in-vivo*	BMT	HSC enrichment	Type II pneumocytes / up to 20%, bronchial epithelium / 4%	Y chromosome FISH, surfactant B mRNA	[31]
Animal, *in-vivo*	Radiation pneumonitis	Whole bone marrow	Type II pneumocytes, bronchial epithelium / up to 20% of type II cells	Y chromosome FISH, surfactant B mRNA	[25]
Animal, *in-vivo*	BMT	Whole bone marrow/EGFP retrovirus	Type II pneumocytes / 1–7%	EGFP, keratin immunostain, surfactant protein B FISH	[33]
Animal, *in-vivo*	BMT and parabiotic animals	HSC	Hematopoietic chimerism but exceedingly rare lung cell types	EGFP	[32]
Animal, *in-vivo*	Bleomycin fibrosis	MSC	Type II pneumocytes / ~1%	Y chromosome FISH	[22]
Animal, *in-vivo*	Radiation fibrosis	MSC or whole bone marrow	Fibroblasts / common	EGFP, Y chromosome FISH, vimentin immunostain	[26]
Animal, *in-vivo*	BMT	Bone marrow, EGFP labeled	Fibroblasts, Type I pneumocyte / occasional to rare	Flow cytometry	[34]
Animal, *in-vitro *and *in-vivo*	Hypoxia-induced pulmonary hypertension	Circulating BM-derived c-kit positive	c-kit positive cells in pulmonary artery vessel wall; In hypoxia, circulating cells generate endothelial and smooth muscle cells *in-vitro*	Flow cytometry and immunohistochemistry	[27]
Animal, in-vivo	Ablative radiation and elastase induced emphysema	GFP + fetal liver	Alveolar epithelium and endothelium; frequency not reported but increased by G-CSF and retinoic acid	Immunohistochemistry for CD45^-^, GFP^+ ^cells	[28]
Animal, in-vivo	Bleomycin fibrosis	Whole marrow GFP^+^	GFP^+ ^type I collagen expressing	Flow cytometry and immunohistochemistry, RT-PCR	[24]
Human, *in-vitro*	Heat shock in cell culture	MSC and SAEC	Cell fusion / common	Immunostaining, microarray	[39]
Animal, *in-vivo* Human, *in-vivo*	OVA-sensitized mouse model Allergen – sensitized asthmatics	CD34 positive, collagen I expressing fibrocytes CD34 positive, collagen I expressing fibrocytes	Myofibroblasts / ? Myofibroblasts / ?	CD34-positive, collagen I, α-smooth muscle actin CD34-positive, collagen I, α-smooth muscle actin	[29]
Human, *in-vivo*	Human heart and lung transplant	Sex-mismatched donor lung or heart	No lung cell types of recipient origin	X and Y chromosome FISH, antibody stain for hematopoeitic cells	[36]
Human, *in-vivo*	Human lung transplant Human BMT	Sex-mismatched donor lung Sex-mismatched donor bone marrow	Bronchial epithelium, type II pneumocytes, glands of recipient origin / 9 – 24% No lung cell types of donor origin	Y chromosome FISH, short tandem repeat PCR Y chromosome FISH, short tandem repeat PCR	[35]
Human, *in-vivo*	Human BMT	Sex-mismatched donor bone marrow	Lung epithelium and endothelium of donor origin / up to 43%	X and Y chromosome FISH, keratin and PECAM immunostain	[38]
Human, *in-vivo*	Human BMT	Sex-mismatched donor bone marrow	No nasal epithelium of donor origin	Y chromosome FISH, cytokeratin immunostain	[37]

BMT = bone marrow transplant (with prior ablation), MSC = mesenchymal stem cells (bone marrow stromal cells, adherent bone marrow cells), EGFP = enhanced green fluorescent protein, HSC = hematopoietic stem cells, FISH = fluoresence in situ hybridization, SAEC = small airway epithelial cells

Transplantation studies in mice can be performed using whole donor bone marrow, the fraction that adheres in culture, termed marrow stromal cells (MSC), or preparations enriched for hematopoietic stem cells (HSC). Whole body irradiation, which may injure lung tissue, is typically used to deplete the host bone marrow. Importantly, lung injury apparently enhances engraftment into lung [22-29]. Whole bone marrow, MSC, or HSC have all been reported to reconstitute lung parenchymal cells. MSC transplantation resulted in collagen I expressing donor cells in the lung [30], and in the presence of bleomycin injury, MSC reportedly generated type I [23] or type II pneumocytes [22]. Transplantation with HSCs yielded up to 20% donor-derived pneumocytes and 4% bronchial epithelial cells [31]. However, other investigators have identified only hematopoeitic chimerism by HSCs [32]. Whole bone marrow infusion generated type II pneumocytes [33], or fibroblasts and type I pneumocytes [34]. Radiation pneumonitis augmented whole bone marrow generation of type II pneumocytes and bronchial epithelial cells [25] or fibroblasts [26]. Bleomycin lung injury enhanced formation of type I collagen-producing cells [24] from whole bone marrow, whereas elastase-induced emphysema stimulated formation of alveolar epithelium and endothelium [28]. Lung injury alone, without bone marrow transplantation, may promote stem cell migration. For example, in the ovalbumin model of asthma, circulating fibrocytes were recruited into bronchial tissue [29], and in a bovine model of hypoxic pulmonary hypertension, cells capable of generating endothelial and smooth muscle cells *in vitro *were found in the circulation [27].

Sex-mismatched lung and bone marrow transplantation in humans provides a natural model for analysis of donor and recipient cell behavior. Bronchial epithelial and gland cells and type II pneumocytes of host origin were reported in one study of lung allografts [35], but not another [36]. After bone marrow transplantation, epithelial cells of donor origin were not detected in the nasal passages [37]. Similar to lung allografts, following bone marrow transplantation, epithelium and endothelium of donor origin were found in one study [38], but not another [35].

**Figure 4:**
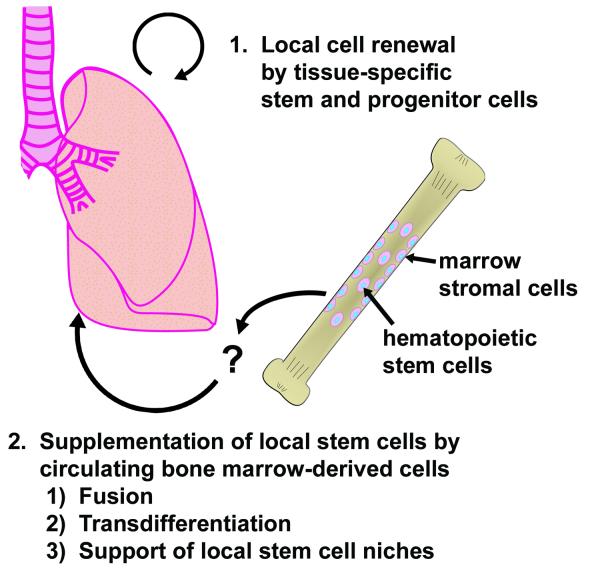
*Evolving view of cell lineages in the lungs*. The functional significance of circulating cells towards lung cell maintenance or tissue repair remains unknown, as does the precise mechanism whereby circulating cells generate lung cell types.

Many questions remain unanswered. The mechanism whereby cells assume lung cell phenotypes remains uncertain. Several studies have demonstrated that cell fusion occurs both *in vitro *and *in vivo*, which likely explains why some of the cells contain both donor and lung cell markers [see [39] for a study of fusion of MSCs and lung epithelium and [40,41] for recent reviews]. Alternatively, cells may reprogram in the lung environment- a concept termed "transdifferentiation", which is defined as the ability of a particular cell from one tissue type to differentiate into a cell type characteristic of another tissue. It has been suggested that many of the events previously attributed to transdifferentiation may actually represent cell fusions, particularly due to the influx of fusion-prone myeloid cells into damaged tissues from the repopulated bone marrow [40]. New, more stringent, criteria have been put forth for demonstration of transdifferentiation [41]. Bone marrow harbors a generalized pluripotent stem cell [42] and the bone marrow cell responsible for lung engraftment has not been identified with certainty. It is possible that rare transdifferentiation events represent migration of a pluripotent bone marrow cell type resembling an embryonic stem or embryonic germ cell still harbored in the adult bone marrow. It remains unknown whether bone marrow cells must transit through an intermediate compartment prior to lung colonization (Figure 4) or whether circulating stem cells can be mobilized from sources other than bone marrow. It is important to note that bone marrow derived cells of typical hematopoietic lineage, chimeric cells created by fusion, or lung cells generated by transdifferentiation may all play a role in lung repair by promoting the local production of stem cells or reparative function of lung-specific cell types. A compelling study suggests that mesenchymal stem cells from bleomycin-resistant mice can mitigate the pro-fibrotic effects of bleomycin in sensitive mice [22], while another study suggests that bone marrow cells actively contribute to the formation of fibrotic tissue [24]. Mitigating or exacerbating roles for bone marrow derived cells in lung repair or fibrosis are not mutually exclusive. The important concepts of whether the lungs' capacity for repair is dependent on circulating cells, and whether exogenously delivered cells can enhance resistance to injury or promote healing, remain unanswered and controversial.

## Lung "stem cell" diseases

Major lung diseases likely involving stem cells and the cellular targets for stem cell therapy are summarized in Table 2. These may be broadly categorized whether they involve stem cell deficiency, hyper-proliferation or possibly, a combination of both. For example, impaired pulmonary endothelial and/or epithelial barrier function may contribute to the pathophysiology of adult respiratory distress syndrome. Mobilization of endogenous endothelial or epithelial stem/progenitor cells or delivery of adult somatic stem cells, embryonic stem cells, or embryonic germ cells may theoretically improve barrier function, supporting the notion of treating a "stem cell deficiency". Similarly, toxic, viral or alloimmune destruction of the bronchiolar epithelium suggests stem cell deficiency in bronchiolitis obliterans. However, fibrotic reactions and scarring in response to epithelial injury can be viewed as fibroblast "stem cell hyper-proliferation". The general concept is that augmentation of stem cells may minimize lung injury, augment repair, or possibly regenerate lost tissue. However, one must also consider that inhibiting excessive growth of stem cells may be a valid therapeutic goal when hyper-proliferation contributes to disease pathophysiology, as in fibrosis, smooth muscle hyperplasia or lung cancer.

**Table 2:** Major lung diseases potentially treatable by stem cell manipulation.

**Disease Category**	Injured, Depleted, or Deranged Cellular Compartment*	**Therapeutic Goals**
Congenital lung hypoplasia Chronic lung disease of prematurity Pulmonary emphysema	Alveolar epithelium, Interstitial fibroblast, Capillary endothelium,	Generate alveolar septa Restore complex three dimensional structure
Neonatal RDS Adult RDS	Alveolar epithelium, Capillary endothelium	Enhance surfactant production Reinforce endothelial and epithelial barriers
Pulmonary fibrosis	Alveolar epithelium, Interstitial fibroblast	Prevent alveolar epithelial loss Inhibit fibroblast proliferation
Asthma	Airway epithelium, Myofibroblasts, Airway smooth muscle	Create an anti-inflammatory environment Inhibit airway wall remodeling Inhibit smooth muscle hypertrophy and hyperplasia
Cystic fibrosis	Airway epithelium	Deliver functional CFTR
Bronchiolitis obliterans	Airway epithelium	Reinforce the epithelium against toxic, viral or immunologic injury
Lung cancer	Epithelium	Detection, monitoring or treatment based on molecular regulation of stem cell proliferation and differentiation

RDS = respiratory distress syndrome, CFTR= cystic fibrosis transmembrane conductance regulator *Each cell type listed in this column is affected in all of the specific conditions listed in the left hand column

## Challenges for lung regenerative medicine

What are the realistic prospects for beneficial stem cell therapy of the lung? First, we must conclusively identify lung diseases/cases/timing in which cell and tissue damage occurs in excess of the capacity for timely endogenous repair. Second, we must establish standardized sources of relevant stem/progenitor cells and methods for their delivery to the appropriate lung sub-compartment. Once delivered, therapeutic cells must home to microscopic sites of need and integrate to serve a beneficial function. There is clearly potential for adverse effects, as exemplified by the propensity of embryonic stem cells to form teratomas when implanted *in vivo *[43]. Major lung diseases potentially addressable by stem cell therapy may pose unique challenges. Reversal of lung developmental anomalies resulting in hypoplasia, or repair of chronic lung disease of prematurity and advanced pulmonary emphysema in adults, will require neogenesis of alveolar septa in which the endogenous "tissue blueprint" never developed, or was completely destroyed. Until we gain a much better understanding of lung tissue morphogenesis, we must rely on stem cells intrinsically "knowing" where to go and "how" to recreate alveolar septal architecture to ultimately restore higher order complex three dimensional relationships amongst alveoli, airways, and vessels. Stem cell therapy to cure cystic fibrosis will require heterologous, or gene corrected autologous, stem cells to colonize the airway, proliferate, and differentiate into columnar cells covering a significant portion of the airway lumen. However, most evidence thus far suggests that cells from the circulation may generate isolated, single airway basal cells. Stem cell therapy to mitigate respiratory distress syndrome (RDS) will require cells capable of restoring alveolar endothelial and epithelial function in the face of evolving injury. Whereas injury is thought to promote stem cell recruitment, the relevant question is whether it can occur quickly enough to meaningfully reverse acute, widespread cellular dysfunction typical of RDS.

## Conclusion

Provocative, but controversial, recent evidence suggests that circulating stem cells may home to the lung. There is great excitement and hope that exogenous and/or mobilized endogenous stem cells may be harnessed to prevent or treat acute and chronic lung diseases and even regenerate abnormally developed or lost tissue. Our understanding of lung stem cells and the regulation of lung morphogenesis is still rudimentary, and the complex, integrated function of multiple cell types underlying normal lung structure and function poses unique challenges. Thus, the therapeutic prospects for stem cell therapy in lungs appear more distant than in some other organs. This realization should stimulate meaningful new studies from the lung research community. Unlike the mythical hero Prometheus, patients with lung disease cannot wait 30,000 years!

## Competing interests

None declared.
